# Properties of GABAergic Neurons Containing Calcium-Permeable Kainate and AMPA-Receptors

**DOI:** 10.3390/life11121309

**Published:** 2021-11-27

**Authors:** Valery Petrovich Zinchenko, Artem Mikhailovich Kosenkov, Sergei Gennadevich Gaidin, Alexander Igorevich Sergeev, Ludmila Petrovna Dolgacheva, Sultan Tuleukhanovich Tuleukhanov

**Affiliations:** 1 Federal Research Center “Pushchino Scientific Center for Biological Research of the Russian Academy of Sciences”, Institute of Cell Biophysics of the Russian Academy of Sciences, 142290 Pushchino, Russia; kosenckov406@yandex.ru (A.M.K.); ser-gajdin@yandex.ru (S.G.G.); ais5885@mail.ru (A.I.S.); dolgacheva@mail.ru (L.P.D.); 2 Laboratory of Biophysics, Chronobiology and Biomedicine, Faculty of Biology and Biotechnology, Al-Farabi Kazakh National University, Almaty 050040, Kazakhstan; sultan.tuleuhanov@kaznu.kz

**Keywords:** GABAergic neurons, GluA2 subunit, GluK1 subunit, Ca^2+^-permeable kainate receptors, Ca^2+^-permeable AMPA receptors, spontaneous synchronous activity

## Abstract

Calcium-permeable kainate and AMPA receptors (CP-KARs and CP-AMPARs), as well as NMDARs, play a pivotal role in plasticity and in regulating neurotransmitter release. Here we visualized in the mature hippocampal neuroglial cultures the neurons expressing CP-AMPARs and CP-KARs. These neurons were visualized by a characteristic fast sustained [Ca^2+^]_i_ increase in response to the agonist of these receptors, domoic acid (DoA), and a selective agonist of GluK1-containing KARs, ATPA. Neurons from both subpopulations are GABAergic. The subpopulation of neurons expressing CP-AMPARs includes a larger percentage of calbindin-positive neurons (39.4 ± 6.0%) than the subpopulation of neurons expressing CP-KARs (14.2 ± 7.5% of CB^+^ neurons). In addition, we have shown for the first time that NH_4_Cl-induced depolarization faster induces an [Ca^2+^]_i_ elevation in GABAergic neurons expressing CP-KARs and CP-AMPARs than in most glutamatergic neurons. CP-AMPARs antagonist, NASPM, increased the amplitude of the DoA-induced Ca^2+^ response in GABAergic neurons expressing CP-KARs, indicating that neurons expressing CP-AMPARs innervate GABAergic neurons expressing CP-KARs. We assume that CP-KARs in inhibitory neurons are involved in the mechanism of outstripping GABA release upon hyperexcitation.

## 1. Introduction

Kainate (KA) and AMPA receptors are ligand-gated channels permeable for Na^+^ and K^+^. However, particular subtypes of KA and AMPA receptors are permeable for Ca^2+^. The Ca^2+^ influx through these receptors can induce neurotransmitter release without the contribution of NMDARs and voltage-gated calcium channels [1]. 

Calcium-permeable KA receptors (CP-KARs) contain unedited GluK1 or GluK2 subunits [2,3]. The excitatory pyramidal neurons express mainly GluK2-containing KARs, whereas GluK1-containing KARs are predominantly expressed by inhibitory interneurons [4,5,6]. Most studies show that GluK1-containing KARs localize on GABAergic neurons in presynaptic terminals [3,7,8,9]. Thus, activation of GluK1-containing kainate receptors may promote the GABA release from interneurons, leading to suppression of neuronal network activity. Contribution of GluK1-expressing GABAergic neurons in the suppression of hyperexcitation was obtained both in in vivo and in vitro experiments. Xu and coauthors demonstrated the neuroprotective effect of a selective GluK1-containing KARs agonist, ATPA, against ischemia-reperfusion-induced neuronal cell death in in vivo studies [10,11]. Furthermore, ATPA suppresses the activity of the neuronal network in cultures [12]. In turn, AMPAR/KAR agonist, DoA [13,14], increases intracellular Ca^2+^ concentration ([Ca^2+^]_i_) in a specific subpopulation of GABAergic neurons, while the activity of all other neurons is suppressed at the same time [15]. It may be suggested that the neuroprotective effects are caused by GABA release induced by the activation of KARs [10,15,16]. However, the properties of these GABAergic neurons and the mechanism of their fast activation are poorly recognized.

Calcium-permeable AMPA receptors (CP-AMPARs) are detected in synapses throughout the brain [17,18]. These receptors are rarely expressed in glutamatergic neurons of mature animals, and their expression occurs mainly after an increase in neuronal activity [19,20]. In contrast, inhibitory interneurons throughout the central nervous system express CP-AMPARs [21,22,23]. However, despite the predominant expression of CP-AMPARs by GABAergic neurons, most studies indicate that activation of these receptors promotes excitation. Excessive activation of CP-AMPARs can cause Ca^2+^-dependent excitotoxic cell death in various pathologies [24,25,26]. Increased expression of CP-AMPARs promotes degeneration of motoneurons and death of pyramidal neurons in CA1 region of the hippocampus after cerebral ischemia [27]. In addition, increased expression of CP-AMPARs in mice leads to seizures and early death at the age of several weeks [28]. In turn, selective CP-AMPAR antagonists prevent neuronal death after ischemia [29] and mechanical injury [30]. 

The various effects CP-AMPARs activation can be explained by different synaptic localization of the receptors. While CP-KARs localize predominantly in presynapses where they regulate GABA release, CP-AMPARs localize in postsynaptic terminals [3], [31,32,33]. Thus, activation of CP-AMPARs may enhance GABA release and have other consequences, including the disturbance of Ca^2+^ homeostasis due to the overactivation of calcium-permeable receptors. The hypothesis that GABAergic neurons expressing CP-AMPARs innervate other GABAergic neurons controlling excitatory neurons can be considered an alternative explanation of the neuroprotective effect of CP-AMPAR antagonists. 

We have previously reported the method of vital identification of CP-AMPARs- and CP-KARs–containing neurons using [Ca^2+^]_i_ imaging [34]. Here we studied the properties and functions of these neurons in rat hippocampal neuroglial culture. Using this method, we revealed the mechanism of outstripping inhibition of glutamatergic neurons by neurons containing CP-KARs. We also performed additional statistical analysis revealing new data regarding the percentage of neurons containing CP-AMPARs and CP-KARs. In addition, we found that CP-AMPARs-containing GABAergic neurons innervate a subpopulation of GABAergic CP-KARs-containing neurons.

## 2. Materials and Methods

All animal studies were performed in accordance with the legal requirements listed in ICB RAS Manual for Working with Laboratory Animals (approved by the Commission on Biosafety and Bioethics of Institute of Cell Biophysics, Protocol No. 57, 30 December 2011), Act 708n (23 August 2010) of the Russian Federation National Ministry of Public Health, which states the rules of laboratory practice for the care and use of laboratory animals, and the Council Directive 2010/63 EU of the European Parliament (22 September 2010) on the protection of animals used for scientific purposes. ICB RAS Animal Facility provided the animals for experiments in accordance with the applications approved by the Commission on Biosafety and Bioethics of Institute of Cell Biophysics (Permission No. 6, 12 December 2017; Permission No. 2, 12 June 2020). Rats were group-housed in the ICB RAS animal facility, with 12 h light-dark cycle and access to food and water ad libitum. All efforts were made to minimize possible pain and discomfort and to reduce the number of used animals.

### 2.1. Cell Culture

Cell co-cultures of hippocampal neurons and astrocytes isolated from the brain of newborn Sprague–Dawley rats (1–3 days old) were prepared as described previously [35,36]. Newborn rats were euthanized (deep inhaled anaesthesia) and then decapitated. The extracted hippocampi were minced with clippers in cold Ca^2+^- and Mg^2+^-free Versene solution. The supernatant was removed with a pipette, and 1% trypsin solution was added. The tissue fragments were trypsinized for 10 min at 37 °C with constant stirring and then washed twice with cold Neurobasal-A medium to inactivate and remove trypsin. The fragments were then gently triturated with a pipette. Non-triturated tissue was removed, and the obtained suspension was centrifuged for 3 min at 2000 rpm. The sedimented cells were resuspended in Neurobasal-A medium supplemented with glutamine (0.5 mM) and B-27 (2%). Cells were seeded on round glass coverslips treated with polyethyleneimine and grown in a CO_2_-incubator (37 °C). The culture medium (2/3 of the volume) was replaced every 3 days. The density of the plated cells was 15.000 cells/sq·cm. Cell cultures at the ages of 12–14 days in vitro (DIV) were used in the experiments. 

### 2.2. [Ca^2+^]_i_ Measurements. Handling of Image Data

The changes of [Ca^2+^]_i_ were evaluated using a fluorescent ratiometric Ca^2+^ sensitive probe, Fura-2 (Molecular probes, Eugene, OR, USA), as described previously [35,37]. The cell cultures were stained for 40 min at 37 °C with freshly prepared Fura-2 AM diluted in HBSS (Hank’s balanced salt solution) to the final concentration 5 µM. Then, the cells were washed with HBSS and incubated for 10–15 min to complete deestherification. To register changes of [Ca^2+^]_i_, we used an inverted motorized fluorescent microscope, Axiovert 200M (Carl Zeiss Microscopy GmbH, Jena, Germany), with a high-speed monochrome CCD-cameraAxioCam HSm (Carl Zeiss Microscopy GmbH, Jena, Germany), and a high-speed light filter replacing system Ludl MAC 5000 (Ludl Electronic Products, Hawthorne, NY, USA). The reagents were added and washed using the perfusion system that provides a perfusion rate 15 mL/min. For Fura-2 excitation and registration, we used the objective Plan-Neofluar 10×/0.3, 21HE filter set (Carl Zeiss, Jena, Germany) with excitation filters BP340/30 and BP387/15, a beam splitter FT-409, and an emission filter BP510/90. Emission of Fura-2 stained cells was recorded upon excitation at the wavelengths of 340 and 387 nm. The frame rate was 1 frame per second. The recording time of one two-channel frame did not exceed 400 ms. The resulting two-channel (when Fura-2 was excited at 340 and 387 nm) time-lapse series of images were processed with ImageJ software. All experiments were carried out at 28–30 °C.

### 2.3. Spontaneous Synchronous Activity

Spontaneous synchronous activity (SSA) is observed throughout the brain and plays a key role in processing neuronal information, brain development, and synaptogenesis [38]. SSA is manifested in the cultures as bursts of action potentials accompanied by oscillations of [Ca^2+^]_i_. It was previously shown that the number of synapses increases significantly during the first two weeks of cell growth in culture. SSA appears in the hippocampal culture a few days after the preparation and lasts for two weeks. Then, the amplitude and frequency of [Ca^2+^]_i_ oscillations significantly decrease due to the GABA-mediated inhibition. However, SSA can be induced in this case by GABA(A) receptor antagonists [37,39]. Oscillations of [Ca^2+^]_i_ during SSA are mediated by the activation of NMDA and predominantly AMPA receptors [39,40]. NBQX, an antagonist of AMPARs/KARs, abolishes the oscillations, while D-AP5, an antagonist of NMDARs, only decreases the amplitude. The SSA induced by removing GABA(A)R-mediated inhibition is rather convenient to study the interaction between cells in the cultures. The difference in agonist-induced Ca^2+^ responses and parameters of SSA between the cultures of 12 and 14 days did not exceed the variation in different experiments of one day. These differences are mainly determined by the ratio of KA and AMPA receptors in neurons. The SSA in the present work was registered using [Ca^2+^]_i_ imaging.

### 2.4. Immunocytochemistry

The immunocytochemical technique was used to identify GABAergic neurons and evaluate the presence of calcium-binding proteins [36,37,41]. Since [Ca^2+^]_i_ measurements and the visualization of the bound antibodies were carried out using different microscopes, the marker grid was plotted on the bottom side of the coverslip with the cell culture to match the images. The recording of Fura-2 fluorescence was performed in one of the grid-bordered areas. After [Ca^2+^]_i_ measurements, the cells in the area were photographed in the phase-contrast mode. Then, cells were fixed and stained with combinations of antibodies against glutamate decarboxylase GAD 65/67, NeuN, neuron-specific enolase (NSE), and calcium-binding proteins (parvalbumin, calbindin, calretinin).

We used freshly prepared 4% paraformaldehyde (PFA) diluted in PBS to fix cells. In the case of anti-GABA antibodies, we added 0.1% glutaraldehyde into the fixative solution to prevent GABA washing from cells during the permeabilization. The cells were incubated with PFA for 20 min and washed three times with ice-cold PBS for 5 min. To permeabilize the cells, we used 0.1% Triton X-100 solution for 15 min. Fixed cells were incubated in 10% donkey or goat serum for 30 min at room temperature to block non-specific antibody binding sites. The cells were then incubated overnight with primary antibodies at 4 °C. We used the next primary antibodies in the present study: rabbit anti-GAD 65/67 antibody (1:500), mouse anti-NeuN antibody (1:300), mouse anti-NSE antibody (1:300), mouse anti-parvalbumin antibody (1:200), mouse anti-calbindin antibody (1:200), mouse anti-calretinin antibody (1:200), rabbit anti-GABA antibody (1:400). All the primary antibodies were diluted in PBS containing 1% of goat/donkey serum. Then, the cells were washed with PBS (3 times for 5 min) and probed with secondary antibodies: donkey anti-rabbit conjugated with Alexa Fluor 647 (1:200), donkey anti-mouse antibodies conjugated with Alexa Fluor 488 (1:200), goat anti-mouse conjugated with Alexa Fluor 633 (1:300), goat anti-rabbit conjugated with Alexa Fluor 555 (1:200). The fluorescence of antibodies was visualized with Leica TCS SP5 inverted confocal microscope using 488 nm argon laser, 543 and 633 nm He-Ne lasers. The registration was conducted at 505–555, 565–600, and 655–700 nm. Then, the confocal images of antibody fluorescence and the series of images of vital fluorescent measurements ([Ca^2+^]_i_ changes) were matched using ImageJ software. This approach makes possible the evaluation of [Ca^2+^]_i_ changes in GABA/GAD 65/67-positive (GABAergic) and GABA/GAD 65/67-negative (glutamatergic) neurons. Neurons were distinguished from astrocytes by synchronous Ca^2+^ activity and staining with mouse monoclonal anti-NeuN or anti-NSE antibodies. Astrocytes did not participate in SSA and did not respond to the KA receptor agonists and KCl (not shown).

### 2.5. Data Analysis

OriginLab Pro 9.1 (OriginLab, Northampton, MA, USA) and Prism GraphPad version 8.0.1 (GraphPad Software, San Diego, CA, USA) software were used for data processing, graph creation, and statistical analysis. All values are given as the mean signal of N cells, or as a typical calcium signal of most cells, or as a signal of individual neurons. Statistical analyses were performed using Student’s test and Kruskal-Wallis test for group comparison. All data were obtained from at least 3 different coverslips with cells chosen from 2–3 independent passages. N—the number of cells analyzed in the individual experiment, n—the number of independent experiments. The number of cells analyzed in one experiment varied from 100 to 200. 

The amplitude of [Ca^2+^]_i_ oscillations or the agonist-induced responses was calculated as the difference between maximum value (averaged by three time points, including the previous/next frame) of the signal (340/387 ratio) during an oscillation or response and basal level of the signal before. For calculations, as a rule, we used the averaged traces of neurons from particular groups. 

### 2.6. Reagents

Domoic acid, ATPA, NASPM hydrochloride, Bicuculline methochloride, NBQX disodium salt, D-AP5 (Tocris Bioscience, Bristol, UK); Neurobasal-A medium, B-27 supplement, Trypsin (2.5%), goat serum (Life Technologies, Grand Island, NY, USA); NH_4_Cl (AppliChem, Darmstadt, Germany); 0.1% polyethylenimine, L-glutamine, paraformaldehyde, rabbit anti-GABA antibodies (Sigma-Aldrich, Saint Louis, MO, USA); Fura-2AM (Molecular Probes, Eugene, OR, USA); rabbit anti-GAD 65/67 antibodies, mouse anti-parvalbumin antibodies, mouse anti-calretinin antibodies, mouse anti-calbindin antibodies, donkey anti-rabbit antibodies conjugated with Alexa Fluor 647, donkey anti-mouse antibodies conjugated with Alexa Fluor 488, goat anti-rabbit antibodies conjugated with Alexa Fluor 555, goat anti-mouse antibodies conjugated with Alexa Fluor 633 (Abcam, Cambridge, UK); mouse anti-NeuN antibody (Santa Cruz Biotechnology, Dallas, TX, USA); mouse anti-NSE (neuron-specific enolase) (Bialexa, Moscow, Russia).

## 3. Results

### 3.1. Identification of Neurons Expressing CP-KARs

**Figure 1:**
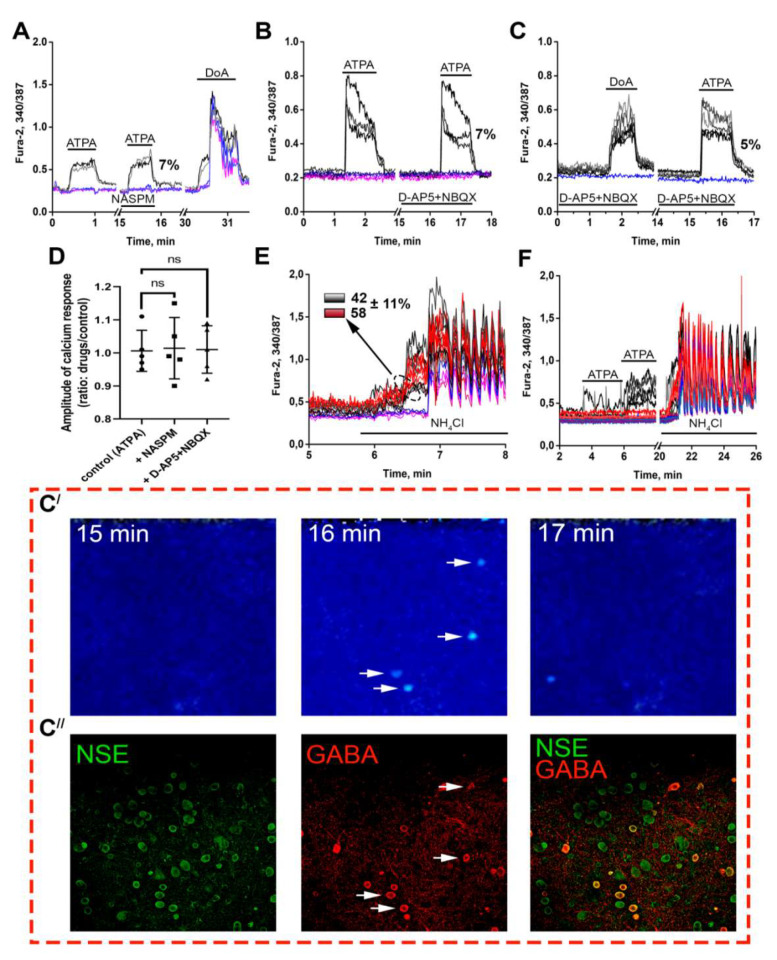
Identification and characteristics of neurons expressing GluK1-containing KARs. (**A**) Selective agonist of GluK1-containing receptors, ATPA (300 nM), induced insensitive to NASPM (30 µM) Ca^2+^ response. DoA (300 nM) evokes a rapid Ca^2+^ response in ATPA-sensitive neurons (black curves). N = 100, *n* = 5. (**B**,**C**) The effects of AMPARs, NMDARs (NBQX, 2 µM; D-AP5, 10 µM) on the ATPA- and DoA-induced [Ca^2+^]_i_ increase. N = 100, *n* = 5. (**D**) Diagram showing the influence of different antagonists on Ca^2+^ response to ATPA application. The ratio of responses to two repeated ATPA applications was used as a control. Kruskal-Wallis test; ns—*p* > 0.9999. (**E**) NH_4_Cl (8 mM) induces an [Ca^2+^]_i_ increase without synchronous oscillations in 16 ± 1% of neurons (black and red curves) earlier than in other neurons (blue and purple curves). N = 100, *n* = 4. (**F**) ATPA (30 and 50 nM) increases [Ca^2+^]_i_ in neurons that faster responded to NH_4_Cl application with an [Ca^2+^]_i_ increase (black curves) N = 100, *n* = 4. (**C′**) Fura-2 340/387 ratio images and (**C″**) and immunostaining of cells in this field with antibodies against NSE and GABA. White arrows indicate ATPA-sensitive neurons. The images in panels C′ and C″ correspond to the experiment presented in Panel C. The values in Panels A–C demonstrate the mean percentage of ATPA-responding neurons from a total number of neurons in a view field.

It was previously shown that selective agonists of GluK1-containing KARs increase [Ca^2+^]_i_ only in certain GABAergic neurons and suppress impulse activity in other neurons in hippocampal cultures [12,15,42]. Figure 1A shows that agonist of GluK1-containing KARs, ATPA, increases [Ca^2+^]_i_ only in a group of neurons (black and grey curves), whereas an antagonist of CP-AMPARs, NASPM, does not change the level of [Ca^2+^]_i_, indicating the absence of CP-AMPARs contribution to the ATPA-induced response (Figure 1A,D). Further application of AMPAR/KAR agonist, DoA (300 nM) (Figure 1A), induces a rapid [Ca^2+^]_i_ increase in ATPA-sensitive neurons (6 ± 1% of neurons) and [Ca^2+^]_i_ oscillations with a delay of 17 ± 4 s in most other neurons. We assumed that ATPA-sensitive neurons are GABAergic, and the observed [Ca^2+^]_i_ rise induces GABA release, suppressing the activity of the innervated neurons. As shown in Figure 1B,D, ATPA-induced [Ca^2+^]_i_ increase is mediated by CP-KARs since the antagonists of NMDARs (D-AP5, 10 μM) and AMPARs (NBQX, 2 μM (selectively blocks AMPARs at this concentration [36])) do not decrease the amplitude of the ATPA-induced Ca^2+^ response. Only ATPA-sensitive neurons react to DoA in the presence of the antagonists (Figure 1C). Both Ca^2+^ responses (DoA-induced in the presence of the antagonists and ATPA-induced) are generated by neurons expressing CP-KARs. We stained the cells with antibodies against GABA and NSE (neuronal marker) to demonstrate that ATPA increases [Ca^2+^]_i_ only in GABAergic neurons expressing CP-KARs. Figure 1C′ shows fluorescent images of the Fura-2-stained cells before ATPA application (15th min of the recording in Figure 1C), in the presence of ATPA (16th min), and after ATPA washing (17th min). We found that ATPA-sensitive neurons (Figure 1C′ 16th min) are GABAergic since they are intensively stained with antibodies against GABA (Figure 1C″ GABA, white arrows). Thus, ATPA can be used to identify GABAergic neurons expressing CP-KA receptors.

We have previously shown that neurons expressing CP-KARs demonstrate increased excitability, possibly due to insufficient GABA(A)R-mediated inhibition [15]. We assumed that such properties allow these neurons to react more quickly to depolarization and application of agonists. To confirm this, we performed the experiments with NH_4_Cl. The ammonium ion is an endogenous toxin that causes acute or chronic hepatic encephalopathy [43,44]. It has been previously shown that ammonium (8 mM) depolarizes neurons in hippocampal cell cultures by 8–10 mV, increases basal [Ca^2+^]_i_ level, and induces high-frequency [Ca^2+^]_i_ oscillations [35,45]. Figure 1E shows that NH_4_Cl increases the basal [Ca^2+^]_i_ level initially only in some neurons (red and black curves), and then these cells generate [Ca^2+^]_i_ oscillations. The remaining neurons react to NH_4_Cl with synchronous [Ca^2+^]_i_ oscillations appearing after a delay (67 ± 15s) (blue and purple curves) without preliminary [Ca^2+^]_i_ rise. ATPA application before the experiment (application not shown) revealed that 42 ± 11% of fast-responding neurons were ATPA-sensitive (Figure 1E, black curves). Even low doses of ATPA increases [Ca^2+^]_i_ in these neurons, indicating their greater excitability (Figure 1F, black curves). Thus, GABAergic neurons expressing GluK1-containing CP-KARs react to NH_4_Cl-induced depolarization faster than most glutamatergic neurons in mature hippocampal cell culture. Therefore, NH_4_Cl-induced Ca^2+^ influx may stimulate GABA release, which suppresses the excitation of glutamatergic neurons.

### 3.2. Identification of Neurons Containing CP-AMPARs

**Figure 2:**
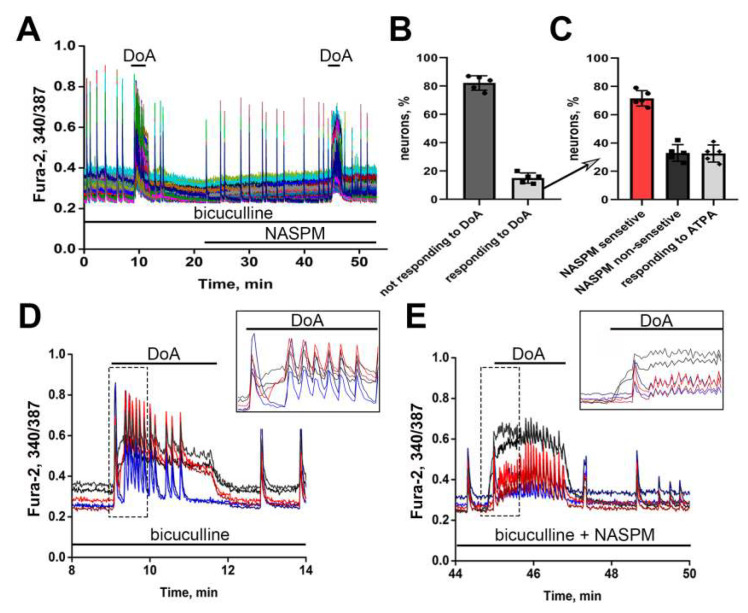
Identification of neurons expressing CP-AMPARs and CP-KARs. (**A**) [Ca^2+^]_i_ changes in 125 neurons in response to 300 nM DoA in the presence of bicuculline (10 µM) in control and in the presence of NASPM (50 µM). (**B**) The percentage of neurons responding and not responding to DoA with a sustained increase of basal [Ca^2+^]_i_. Mean ± SD, *n* = 4. (**C**) The percentage of NASPM-sensitive and NASPM-insensitive neurons among those which respond to DoA with a sustained [Ca^2+^]_i_ increase, and the percentage of NASPM-insensitive neurons responding to ATPA. (**D**,**E**) Magnified calcium signals (from Panel A) during the first seconds after DoA application in control (**D**) and in the presence of NASPM (E). On all presented graphs, the red color corresponds to NASPM-sensitive neurons expressing CP-AMPARs, while the black color corresponds to ATPA-sensitive neurons expressing CP-KARs; the blue color corresponds to glutamatergic neurons. N = 125, *n* = 4.

To visualize neurons expressing CP-AMPARs and CP-KARs in the same experiment, we used low doses of DoA (300 nM). DoA activates KARs and AMPARs [13,14,46] and causes the influx of Ca^2+^ into neurons through CP-KA and CP-AMPA receptors. The experiment was performed in the presence of bicuculline to abolish GABA(A)R-mediated inhibition and induce SSA [12,37,40,47,48]. Applications of DoA in the absence and the presence of NASPM (a CP-AMPAR antagonist) were made to distinguish neurons expressing CP-KARs from neurons containing CP-AMPARs (Figure 2A). A short-term application of 300 nM DoA causes a rapid, sustained increase in basal [Ca^2+^]_i_ in some neurons (red and black curves in Figure 2D). In all other neurons, DoA increases the frequency of [Ca^2+^]_i_ oscillations (blue curves in Figure 2D) within seconds (15 s in this experiment). Application of DoA in the presence of NASPM revealed that the neurons reacted to DoA with a sustained increase in basal [Ca^2+^]_i_ can be divided into two subpopulations. NASPM did not suppress (even increased) the fast DoA-induced [Ca^2+^]_i_ rise in neurons of the first subpopulation (Figure 2E, black curves) and suppressed the increase of basal [Ca^2+^]_i_ in neurons of the second subpopulation (Figure 2E red curves) that reacted to DoA with a fast sustained [Ca^2+^]_i_ increase in the control experiment (Figure 2D, red curves). Therefore, the second subpopulation can be attributed to neurons expressing CP-AMPARs. Figure 2B shows that 15 ± 3.6% of neurons respond to DoA with a sustained increase of basal [Ca^2+^]_i_. NASPM-sensitive neurons accounted for 71.6 ± 5.5% of DoA-responding neurons (Figure 2C). The number of NASPM-insensitive neurons coincided with the number of neurons that responded to ATPA, so they can be attributed to neurons containing GluK1 subunit.

As previously shown, the abolishment of GABA(A)R-mediated inhibition by bicuculline causes postsynaptic membrane depolarization and activates AMPARs and NMDARs, thus inducing regular spontaneous [Ca^2+^]_i_ oscillations [12,39]. However, DoA-activated kainate receptors are insensitive to bicuculline-induced postsynaptic membrane depolarization [42], implying the absence of GluK1-containing CP-KARs in the postsynaptic membrane of these neurons. Figure 1A and Figure 2C show that NASPM-insensitive neurons responding to DoA application with a sustained [Ca^2+^]_i_ increase also respond to ATPA, indicating the presence of GluK1-containing CP-KARs. Thus, the activation of DoA-sensitive neurons probably stimulates GABA release, which suppresses calcium response in glutamatergic neurons in the absence of the GABA(A)R antagonist. Thus, DoA- and ATPA-induced Ca^2+^ responses make it possible to visualize GABAergic neurons expressing CP-KARs and CP-AMPARs. The CP-KARs and CP-AMPARs are expressed in different neurons and in the different terminals: CP-AMPARs in postsynaptic terminals and CP-KARs in presynaptic.

### 3.3. Neurons Expressing CP-AMPARs Are GABAergic

**Figure 3:**
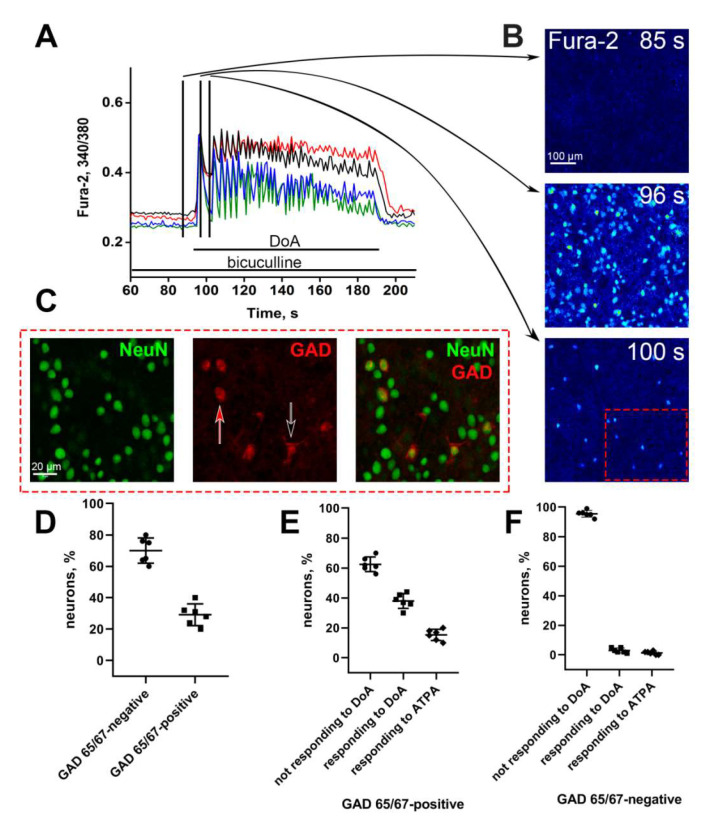
Vital Ca^2+^ imaging and further immunostaining of neurons expressing CP-KARs and CP-AMPARs. (**A**) Calcium responses of representative neurons from each of the four subpopulations to DoA application (300 nM) in the presence of bicuculline (10 µM). The black curve—neurons expressing CP-KARs (NASPM-insensitive neurons); the red curve—neurons expressing CP-AMPARs (NASPM-sensitive neurons); the green and blue curves correspond to glutamatergic neurons. The vertical lines in Figure 3A show the moments corresponding to the images shown on the right panel. (**B**) Fluorescent images of cells stained with Fura-2. The upper image shows cells without any exposures. The middle image corresponds to the maximum of the calcium pulse (bright cells). Dark cells – astrocytes. The bottom image shows neurons (single bright cells) expressing CP-KARs and CP-AMPARs (neurons that respond faster to DoA). The numbers in the pictures show the time since the experiment beginning. (**C**) Immunostaining of neurons with antibodies against GAD 65/67 and NeuN. The presented images correspond to the area bordered with a red dotted square frame at the bottom of Figure 3B. The red arrow indicates neurons expressing CP-AMPARs; the black arrow indicates neurons expressing CP-KARs. (**D**) The percentage of GAD65/67-positive and GAD 65/67-negative neurons. (**E**,**F**). The percentage of neurons responding and not responding to DoA and ATPA in the groups of GAD 65/67-positive and GAD 65/67-negative neurons. Results are presented as the mean ± SD. N = 200, *n* = 6.

To prove that all neurons responding to DoA with a sustained [Ca^2+^]_i_ increase (red and black curves in Figure 2D) are GABAergic, we stained the cultures with antibodies against GAD 65/67 (a marker of GABAergic neurons) and the neuronal marker, NeuN (Figure 3C). Before immunostaining, we identified these neurons by the high level of [Ca^2+^]_i_ after 100 s DoA exposure (Figure 3B, the bottom image).

Immunostaining revealed that the neurons responding to DoA with a sustained [Ca^2+^]_i_ increase mainly belong to the GABAergic (Figure 3C,F). In turn, the neurons that reacted to DoA with the delayed increase in SSA frequency (blue and green curves in Figure 2D,E and Figure 3A) were not stained with antibodies against GAD65/67 and can be attributed to the glutamatergic neurons. Thus, we showed that both subpopulations of neurons responding to DoA and NH_4_Cl with a sustained [Ca^2+^]_i_ increase are GABAergic. Neurons of one subpopulation express CP-KARs, and the other one-CP-AMPARs. In the hippocampal culture, 29 ± 4% of neurons were stained with anti-GAD65/67 antibodies (Figure 3D); DoA-sensitive GABAergic neurons account for approximately 39 ± 2% of GAD 65/67-positive cells (Figure 3E). Among them, 32.6 ± 6% of neurons express CP-KARs (Figure 2C). The fraction of GAD65/67-negative cells does not practically include neurons that express CP-KARs and CP-AMPARs (Figure 3F).

### 3.4. Calcium-Binding Proteins in Neurons Expressing CP-AMPARs and CP-KARs

**Figure 4:**
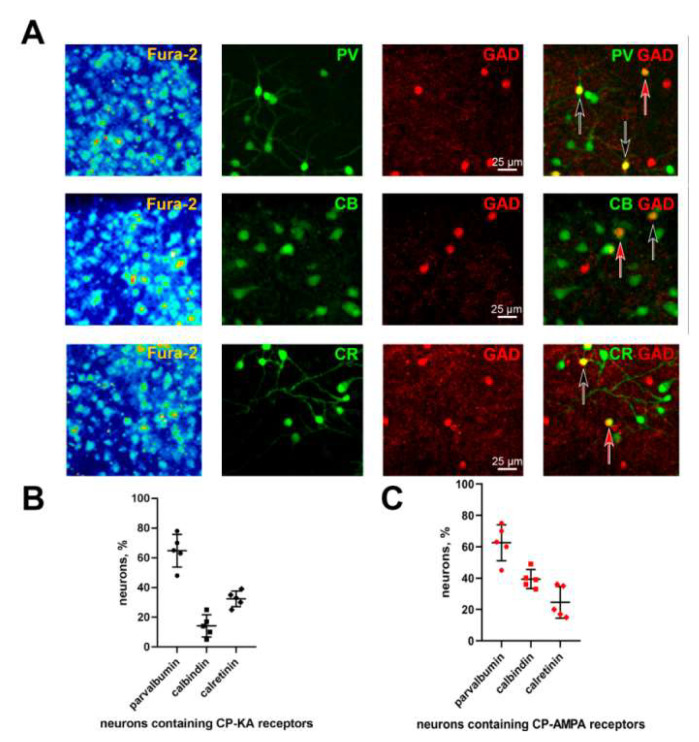
The presence of the calcium-binding proteins in neurons expressing CP-AMPARs and CP-KARs. (**A**) Immunocytochemical staining of neurons with antibodies against parvalbumin (PV), calbindin (CB), calretinin (CR), and GAD 65/67. The left column shows images of neurons loaded with Fura-2. The second column shows cells stained with antibodies against CBPs; the third column shows cells stained with antibodies against GAD 65/67; the fourth column merges Columns 2 and 3. The red arrow indicates a neuron expressing CP-AMPARs; the black arrow indicates neurons expressing CP-KARs. (**B**,**C**) The percentage of PV^+^, CB^+^, and CR^+^ neurons among GAD 65/67-positive neurons expressing CP-KARs (B) and CP-AMPARs (C). The results are presented as the mean ± SD, *n* = 4.

GABAergic neurons can express various calcium-binding proteins (CBPs) [49,50]. Interestingly, some CBPs are exclusively expressed by GABAergic neurons and are considered their specific markers [49,51]. To determine the presence of CBPs in GABAergic neurons expressing CP-KARs or CP-AMRARs, we used double staining with antibodies against parvalbumin (PV) and GAD 65/67 (Figure 4A, upper line); calbindin (CB) and GAD 65/67 (Figure 4A, middle line); calretinin (CR) and GAD 65/67 (Figure 4A, bottom line). 

Neurons expressing CP-AMPARs and CP-KARs were identified by the sensitivity to NASPM and by the presence of ATPA-induced Ca^2+^-response described in Section 3.1 and Section 3.2 (Figure 1 and Figure 2). Figure 4B,C demonstrate the percentage of the PV^+^, CB^+^, and CR^+^ GAD 65/67-positive neurons expressing CP-KARs (Figure 4B) and CP-AMPARs (Figure 4C). Figures show that both groups of neurons include a significant number of parvalbumin-containing cells (62.6 ± 11.5% for CP-AMPARs-containing and 64.8 ± 11.0% for CP-KARs-containing) and a small number of calretinin-containing cells (24.6 ± 10.1% for CP-AMPARs-containing and 32.4 ± 5.3% for CP-KARs-containing). However, the subpopulation of neurons expressing CP-AMPARs includes more CB^+^ neurons (39.4 ± 6.0%) than the subpopulation of neurons expressing CP-KARs (14.2 ± 7.5% of CB^+^ neurons). Thus, the presence of calbindin in neurons expressing CP-AMPARs may partially explain the slower calcium response of these neurons to DoA.

### 3.5. GABAergic Neurons Expressing CP-AMPARs Inhibit GABAergic Neurons Expressing CP-KARs

**Figure 5:**
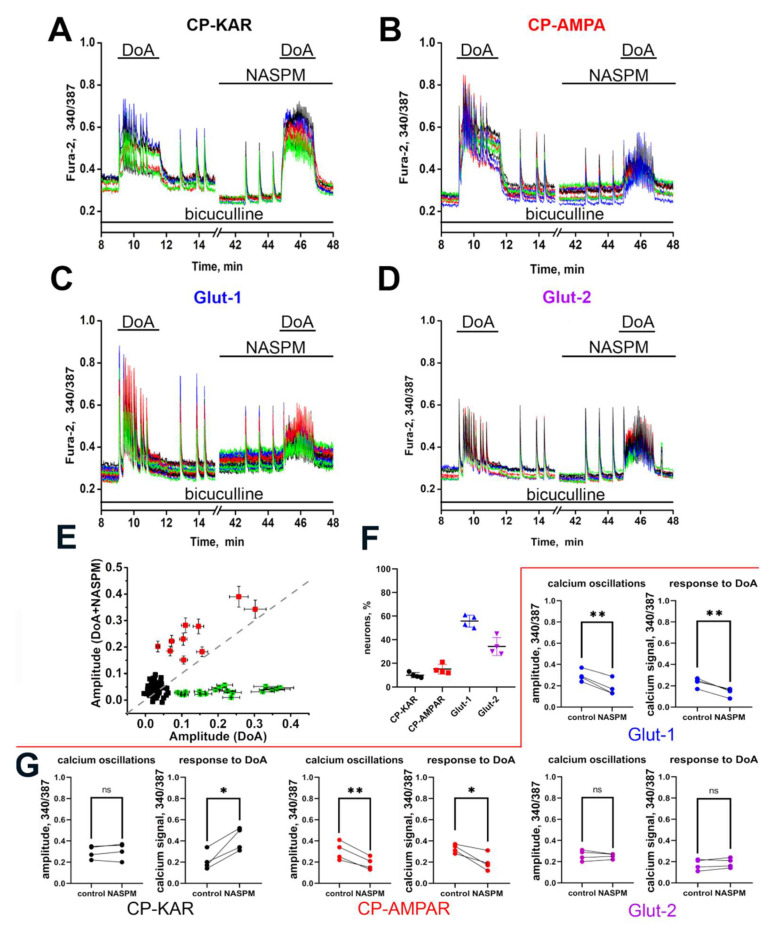
(**A**–**D**) The effect of NASPM on the activity of neurons from four subpopulations. [Ca^2+^]_i_ changes in neurons of different subpopulations from Fig. 2A. Calcium response to DoA and [Ca^2+^]_i_ oscillations in control (left parts) and in the presence of NASPM (right parts) in four subpopulations of neurons: GABAergic neurons are marked as CP-KAR (7 cells) and CP-AMPAR (10 cells); glutamatergic neurons are marked as Glut-1 (31 cells) and Glut-2 (19 cells). (**E**) The effects of NASPM on the amplitude of DoA-induced Ca^2+^ responses. Green and red markers—the cells responded to DoA; black markers—neurons that do not respond to DoA application. Bars show the 10% error. (**F**) The percentage of neurons from each subpopulation. (N = 125, *n* = 4). (**G**) Diagrams showing the amplitudes of [Ca^2+^]_i_ oscillations (left) and the amplitude of DoA-induced calcium responses (right) in control and in the presence of NASPM for each neuronal subpopulation. Paired t-test. CP-KAR: ns, *p* = 0.3677, * *p* = 0.0170; CP-AMPAR: ** *p* = 0.0062, * *p* = 0.0278; Glut-1: ** *p* = 0.0041, ** *p* = 0,0029; Glut-2: ns, *p* = 0.6238, ns, *p* = 0.2152.

Fluorescent Ca^2+^ imaging allows to record changes in the activity of target (innervated) neurons in response to changes in the activity of GABAergic neurons [15,42]. To identify neurons innervated by GABAergic neurons expressing CP-KARs and CP-AMRARs, we analyzed the amplitude and frequency of SSA and DoA-induced calcium responses in control and in the presence of NASPM (Figure 5A,D) in four neuronal subpopulations from the experiment shown in Figure 2A. The percentage of neurons in each subpopulation is demonstrated in Figure 5F. As can be seen (Figure 5E, DoA increases basal [Ca^2+^]_i_ in 28 neurons (green and red markers) out of 125 in a view field in control. In the presence of NASPM, DoA suppresses the signal in 17 neurons and increases in 9 neurons out of these 28. 

In the presence of NASPM, the amplitude of [Ca^2+^]_i_ oscillations after DoA washout did not change in CP-KARs-expressing GABAergic neurons (Figure 5A, CP-KAR). This finding confirms the lack of CP-AMPARs in these neurons. However, a significant increase in the amplitude of DoA-induced Ca^2+^ response in neurons of this subpopulation in the presence of NASPM indicates that these neurons are innervated by GABAergic neurons containing CP-AMPARs. The amplitude of the DoA-induced Ca^2+^ response in the presence of NASPM did not increase in any other neurons. It seems that NASPM inhibits Ca^2+^-influx and Ca^2+^-dependent GABA release in interneurons expressing CP-AMPARs, thus abolishing GABA-mediated inhibition of neurons expressing CP-KARs. The amplitude of [Ca^2+^]_i_ oscillations after DoA washout decreases in NASPM-sensitive GABAergic neurons in the presence of NASPM by 29 ± 2% (Figure 5B, CP-AMPAR), indicating that these receptors are involved in the generation of Ca^2+^ pulses during SSA. A significant decrease in amplitude of DoA-induced [Ca^2+^]_i_ response is observed in neurons of this subpopulation in the presence of NASPM. This effect probably occurs due to direct inhibition of the fast [Ca^2+^]_i_ increase (see Figure 2E).

The amplitude of [Ca^2+^]_i_ oscillations in one subpopulation of glutamatergic neurons (Glut-1) depended on the presence of NASPM and did not depend in the second one (Glut-2) (Figure 5C,D). The amplitude of SSA decreased in glutamatergic neurons (Glut-1) in the presence of NASPM. Since glutamatergic neurons do not virtually express CP-KARS and CP-AMPARs (Figure 3F), we can assume that Glut-1 neurons are innervated by GABAergic neurons expressing CP-KARs, which activity significantly increases at this time (Figure 5A, CP-KAR). The SSA amplitude does not change in glutamatergic neurons (Glut-2) in the presence of NASPM. The amplitude of DoA-induced calcium oscillations also did not change in the presence of NASPM, indicating that this subpopulation of neurons is not innervated by GABAergic neurons expressing CP-AMRARs or CP-KARs. Thus, this experiment suggests that GABAergic neurons expressing CP-AMPARs innervate GABAergic neurons expressing CP-KARs, which, in turn, control the numerous subpopulation of glutamatergic neurons. 

## 4. Discussion

Two subpopulations of GABAergic neurons expressing CP-KARs and CP-AMPARs were detected in rat hippocampal cell cultures on 12–14 days in vitro (DIV). The neurons expressing CP-KARs were identified by the [Ca^2+^]_i_ increase in response to ATPA. ATPA-induced Ca^2+^ response was insensitive to antagonists of NMDA and AMPA receptors, indicating that ATPA-induced depolarization is not enough to activate NMDARs. Neurons expressing CP-AMPARs were identified by the sensitivity of DoA-induced [Ca^2+^]_i_ increase to the antagonist of CP-AMPA receptors, NASPM. We found in the present work that CP-KARs and CP-AMRARs are localized mainly on various subpopulations of GABAergic neurons. The results are consistent and complement the data, according to which GluK1-containing KA receptors are mainly expressed in a certain subpopulation of GABAergic neurons in the hippocampus [4,5,6]. It was also shown that kainate causes rapid Ca^2+^ influx and [Ca^2+^]_i_ increase in distinct subpopulations of GABAergic neurons [52]. Agonists of CP-KA and CP-AMPA receptors selectively increase [Ca^2+^]_i_ in these neurons, leading to the GABA release and inhibition of other different neurons in the network [31]. This inhibition is largely abolished by the antagonist of GABA(A) receptors, bicuculline [15]. 

Unlike ATPA, DoA induces a sustained increase of basal [Ca^2+^]_i_ in both CP-KARs- and CP-AMPARs-expressing GABAergic neurons. In turn, [Ca^2+^]_i_ oscillations in glutamatergic neurons (Glut-1 and Glut-2) appeared with a delay and were not followed by a sustained increase in basal [Ca^2+^]_i_. Early DoA-induced [Ca^2+^]_i_ rise is not accompanied by neuronal excitation, and [Ca^2+^]_i_ oscillations appeared in all neurons only after 60 s of DoA exposure. Considering the existence of voltage thresholds for the spreading of depolarizing stimulus, it may be suggested that the currents mediated by CP-KARs and CP-AMPARs are insufficient to excite GABAergic neurons but sufficient to trigger the GABA release. Slower Ca^2+^ removal in these neurons may promote faster Ca^2+^ accumulation in the cytosol, potentiating GABA release. 

The massive GABA release due to the faster response of GABAergic neurons expressing CP-AMPARs and CP-KARs may explain the delayed response in Glut-1 and Glut-2 neurons. This assumption also explains the delay of response in most neurons after NH_4_Cl application (Figure 1E). As previously shown [16], DoA-sensitive neurons are more excitable than other neurons, probably due to insufficient GABA(A)R-mediated inhibition. This feature probably allows GABAergic neurons to respond to an excitatory stimulus earlier compared to other neurons (glutamatergic) and release GABA, thus suppressing the activity of the innervated neurons. In addition, the higher excitability of GABAergic neurons may be explained by the altered KCC2 expression, affecting Cl^−^ gradient and the response of neurons to GABA [53,54]. 

It is known that GABAergic neurons are subdivided into several subtypes that differ in the expression of calcium-binding proteins [49,50,55]. The role of these proteins is actively discussed [56,57]. Calcium-binding proteins protect GABAergic neurons against a global [Ca^2+^]_i_ increase under oxygen-glucose deprivation [41]. Here we showed the correlation between the rate of [Ca^2+^]_i_ increase and the presence of CBPs in neurons. We found that a subpopulation of neurons expressing CP-AMPARs includes a larger percentage of CB^+^ neurons (39.4 ± 6.0%) than the subpopulation of neurons expressing CP-KARs (14.2 ± 7.5% of CB^+^ neurons). The presence of calbindin in neurons expressing CP-AMPARs may explain the slower calcium response of these neurons to DoA [34]. Thus, a subpopulation of GABAergic neurons expressing CP-AMPARs may include a subtype of neurons expressing calbindin. Fast-binding Ca^2+^ proteins, such as CB and CR, decrease the rate of [Ca^2+^]_i_ rise and limit the amplitude of the Ca^2+^ signal, thus slowing or preventing GABA release at low frequencies of [Ca^2+^]_i_ oscillations. However, when the cell is hyperexcited, the average rate of Ca^2+^ entry into cytosol exceeds the pumping rate. Repletion of the intracellular Ca^2+^ buffers will increase the amplitude of [Ca^2+^]_i_ changes and, accordingly, increase the intensity of neurotransmission. This mechanism can be used to suppress the activity of target neurons under hyperexcitation. Calcium-binding proteins can also protect neurons from damage under oxygen and oxygen-glucose deprivation [41]. 

We analyzed the [Ca^2+^]_i_ changes of hundreds of neurons to establish interaction between neuronal subpopulations. We showed that inhibition of CP-AMRARs by NASPM causes overexcitation of GABAergic neurons expressing CP-KARs (Figure 5A), pointing out that GABAergic neurons expressing CP-AMPARs inhibit the activity of GABAergic neurons expressing CP-KARs. This mechanism may be considered negative feedback, which aims to suppress the increased activity of GABAergic neurons expressing CP-KARs. 

A decrease in the activity of some glutamatergic neurons in the presence of NASPM correlates with increased activity of neurons expressing CP-KARs (Figure 5A,C; Glut-1), thus indicating the innervation of these glutamatergic neurons by inhibitory neurons expressing CP-KARs. We also found a subpopulation of glutamatergic neurons, which do not respond to the antagonist of CP-AMPARs (Figure 5D, Glut-2). Probably, these neurons do not contain CP-AMPARs and are not innervated by NASPM-sensitive neurons. This finding agrees with the data of Wondolowski and colleagues [58], showing that interneurons expressing KARs innervate only a specific subpopulation of neurons. The inhibition of cholecystokinin-releasing interneurons innervating pyramidal cells by GABAergic neurons with presynaptic KARs also has been shown [59]. Our data suggest that GABAergic neurons expressing CP-KARs and CP-AMPARs can suppress hyperexcitation of other neurons due to rapid, sustained increase of [Ca^2+^]_i_ followed by GABA release. Thus, during the development of neurons in culture for 14 days, the neuronal network self-organizes, and interactions between neurons expressing CP-AMPARs, and CP-KARs are formed. There is no direct evidence for the presence of such mechanisms in vivo, although indirect experiments indicate this possibility [24,25,26,27,28,29]. The earlier response of GABAergic neurons expressing GluK1-containing KARs to depolarization may be considered as an inhibition mechanism in neuronal networks.

## 5. Conclusions

We showed a new mechanism of the firing activity regulation in hippocampal cell culture by GABAergic neurons expressing CP-KARs and CP-AMPARs. Two different subpopulations of GABAergic neurons were identified. One subpopulation contains CP-KARs, and the other one contains CP-AMPARs. CP-KARs and CP-AMPARs agonists cause a rapid [Ca^2+^]_i_ increase followed by GABA release in these neurons, thus suppressing the activity of other neurons. In addition, these GABAergic neurons respond faster than other neurons to NH_4_Cl-induced depolarization. The innervation of GABAergic neurons expressing CP-KARs by GABAergic neurons expressing CP-AMPARs explains the excitatory effect of agonists and the neuroprotective effect of antagonists of CP-AMPARs.

The obtained data suggest that GABAergic neurons expressing CP-KARs and CP-AMPARs can inhibit hyperexcitation of other neurons due to faster reaction to an excitatory stimulus leading to GABA secretion.

## Data Availability

All data are provided in full in the results section of this paper. The authors confirm that all data underlying the finding are available and will be shared with the research community upon request.
